# Involvement of RhoA/ROCK Signaling Pathway in Methamphetamine-Induced Blood-Brain Barrier Disruption

**DOI:** 10.3390/biom15030340

**Published:** 2025-02-27

**Authors:** Jong Su Hwang, Tam Thuy Lu Vo, Mikyung Kim, Eun Hye Cha, Kyo Cheol Mun, Eunyoung Ha, Ji Hae Seo

**Affiliations:** Department of Biochemistry, School of Medicine, Keimyung University, Daegu 42601, Republic of Korea; z_zone8863@naver.com (J.S.H.); volutam@gmail.com (T.T.L.V.); cjstk2227@nate.com (M.K.); chaeunhye_7@naver.com (E.H.C.); mun@dsmc.or.kr (K.C.M.); hanne.md@gmail.com (E.H.)

**Keywords:** methamphetamine, blood–brain barrier, RhoA/ROCK signaling pathway, primary human brain microvascular endothelial cells, Y-27632

## Abstract

Methamphetamine (METH) is a powerful addictive psychostimulant that gives rise to severe abusers worldwide. While many studies have reported on the neurotoxicity of METH, blood–brain barrier (BBB) dysfunction has recently attracted attention as an essential target in METH-induced pathological changes in the brain. However, its mechanism has not been fully understood. We found that METH increased paracellular permeability and decreased vascular integrity through FITC–dextran and trans-endothelial electrical resistance (TEER) assay in primary human brain endothelial cells (HBMECs). Also, redistribution of tight junction proteins (zonula occluden-1 and claudin-5) and reorganization of F-actin cytoskeleton were observed in METH-exposed HBMECs. To determine the mechanism of METH-induced BBB disruption, the RhoA/ROCK signaling pathway was examined in METH-treated HBMECs. METH-activated RhoA, followed by an increase in the phosphorylation of downstream effectors, myosin light chain (MLC) and cofilin, occurs in HBMECs. Pretreatment with ROCK inhibitors Y-27632 and fasudil reduced the METH-induced increase in phosphorylation of MLC and cofilin, preventing METH-induced redistribution of junction proteins and F-actin cytoskeletal reorganization. Moreover, METH-induced BBB leakage was alleviated by ROCK inhibitors in vitro and in vivo. Taken together, these results suggest that METH induces BBB dysfunction by activating the RhoA/ROCK signaling pathway, which results in the redistribution of junction proteins via F-actin cytoskeletal reorganization.

## 1. Introduction

Methamphetamine (METH) is a highly addictive psychostimulant that causes significant physical and mental health problems due to its high potential for abuse. It is well known that METH leads to permanent brain damage via disruption of structure and function of dopaminergic neurons in various brain regions, including the cortex, hippocampus, and striatum areas, which are commonly affected in neurodegenerative diseases [1,2]. Extensive studies have reported that METH-induced neurotoxicity activates non-neuronal cells, such as microglia, astrocytes, and endothelial cells, resulting in irreversible negative effects through neuroinflammatory responses [3,4,5]. Moreover, recent studies have revealed that METH-related vascular dysfunction contributes to numerous brain diseases [6,7,8].

The blood–brain barrier (BBB) plays a critical role in maintaining the brain environment by serving as both a physical and a chemical barrier [9]. The BBB strictly restricts the entry of toxic substances such as compounds, bacteria, and hydrophilic molecules, but allows hydrophobic molecules, amino acids, and glucose, which are essential for the survive for neuronal cells in the brain, to pass through [10]. It is well established that BBB integrity is preserved by adjacent brain endothelial cells, which are tightly linked by tight junctions (TJs) and adherens junctions (AJs) that connect to the cytoskeleton [11,12]. BBB injury has been reported in various central nervous system (CNS) diseases, including traumatic brain injury (TBI), stroke, and Alzheimer’s disease [13,14,15]. Several studies have recently suggested that METH disrupts BBB integrity, resulting in an increase in BBB permeability [16,17,18,19]. As a result, METH-induced BBB dysfunction has emerged as a crucial therapeutic target for treating METH abuse [16,20,21]. However, the molecular mechanisms underlying METH-induced breakdown of the BBB are still unidentified.

A current investigation reported that the mechanism of METH-induced BBB disruption is related to the RhoA/ROCK signaling pathway [22]. The RhoA/ROCK pathway is a major signal transduction pathway that regulates cell morphology, migration, and actin cytoskeletal organization [23]. Numerous recent studies have shown that the RhoA/ROCK signaling pathway is closely related to the regulation of TJ proteins in brain capillary endothelial cells [24,25,26]. The small GTP-binding protein RhoA activates ROCK and subsequently phosphorylates downstream effector molecules: MLC (myosin light chain) and cofilin (actin depolymerizing factor, ADF) [27]. ROCK is a critical downstream target of Rho signaling, which can directly phosphorylate MLC and further LIM kinase (LIMK), thereby phosphorylating cofilin [28]. Phosphorylation of MLC induces actomyosin contraction, resulting in the formation of stress fibers, while phosphorylated cofilin becomes inactive, and also contributes to the cytoskeletal reorganization in consequence of severe elongation of actin filaments, leading to an increase in barrier permeability through the modification of TJ proteins in brain endothelial cells [29,30].

Y-27632 and fasudil are selective ROCK1/2 inhibitors, which have been widely used as therapeutic agents in numerous brain damages such as cerebral ischemia injury, cerebral vascular disorders, and neurodegenerative disorders [29,31,32,33,34,35]. Many studies have demonstrated that ROCK inhibitors improve neurological function through the reduction in neuronal apoptosis and the increase in neurite growth [36,37,38]. In addition, several studies have reported that inhibition of ROCK improves vascular function through cytoskeletal reorganization mediated by the activation of the RhoA/ROCK pathway [39,40,41].

A proper in vitro mimic BBB model is critical for studying the effect of compounds on BBB functions. One of the most common in vitro BBB models is culturing monolayers of animal brain endothelial cells. However, animal brain endothelial cells do not accurately represent the human BBB due to significant molecular differences between human and animal BBBs [42]. Moreover, over 80% of drug candidates that demonstrate efficacy in animal models fail during clinical trials [43]. Therefore, in vitro mimic BBB models based on human brain endothelial cells offer a more physiologically relevant and precise platform for studying human BBB functions. Among the human-derived brain capillary endothelial cells, primary human brain microvascular endothelial cells (HBMECs) have been characterized as the most suitable cell type for in vitro BBB models, particularly regarding to BBB integrity [44].

In the present study, we demonstrated the fundamental mechanism underlying METH-induced BBB dysfunction using HBMECs as an in vitro BBB model. Our findings showed that METH increased the phosphorylation of downstream effector molecules in the RhoA/ROCK signaling pathway, leading to impaired BBB integrity and function in primary HBMECs.

## 2. Materials and Methods

### 2.1. Materials

Y-27632(A11001) and Fasudil(A10381) were purchased from AdooQ BioScience (Irvine, CA, USA). Methamphetamine (METH) was obtained from the Ministry of Food and Drug Safety (Cheongju, Korea). Cell counting kit-8 (CCK-8) was purchased from Dojindo (Kumamoto, Japan). The antibodies were purchased from the following sources: ZO-1 (#40-2200) and occludin (#33-1500) were from Invitrogen (Carlsbad, CA, USA), VE-cadherin (ab33168) was from Abcam (Cambridge, UK), and claudin-5 (sc-374221) and β-actin (sc-47778) were from Santa Cruz Biotechnology (Dallas, TX, USA).

### 2.2. Cell Culture

Primary human brain microvascular endothelial cells (HBMECs) were purchased from Cell Systems (Kirkland, WA, USA) and cultured in EGM-2 (CC-3162, Lonza, Walkersville, MD, USA) supplemented with 2% FBS (Gibco, New York, NY, USA) and 1% penicillin streptomycin. HBMECs were maintained in type-1 collagen-coated tissue culture polystyrene flasks (T-flask) at 37 °C and a 5% CO_2_ incubator.

### 2.3. Cell Viability Assay

Cell viability was assessed by CCK-8 assay. Briefly, HBMECs plated in collagen-coated 96-well plates (2 × 10^4^ cells per well) were treated with METH (0.1, 1.0, and 5.0 mM) for 24 h. Then, CCK-8 reagent was added into the each well for 1 h at 37 °C in a 5% CO_2_ incubator [45]. The absorbance was determined at 452 nm using a microplate reader (Tecan, Männedorf, Switzerland). Percentage of viability was calculated against the untreated cell.

### 2.4. Trans-Endothelial Electrical Resistance (TEER)

TEER was measured with using STX2/chopstic electrodes coupled to an EVOM2 resistance meter (World Precision Instruments, Sarasota, FL, USA). The confluence of plated HBMECs was determined for each TEER reading. Once the cells reached confluence, cells were pretreated with Y-27632 (10 uM) for 30 min before METH (1 mM) treatment for 24 h. Untreated samples were designated as the control sample. Three independent experiments with triplicates for each treatment were performed. The TEER values were calculated by subtracting the resistance of treated wells from that of the coated well without cells and correcting to the surface area [46].

### 2.5. In Vitro Permeability Assay

The in vitro permeability assay was performed using transwell cell culture inserts (0.4 μm pore size, 6.5 mm diameter; Corning). HBMECs were plated onto the collagen-coated membrane of the inserts and then maintained until 100% confluency. To measure the paracellular permeability, fluorescein isothiocyanate (FITC)–conjugated dextran (70 kDa; Sigma-Aldrich, St. Louis, MO, USA) was added into the upper compartment of the insert at a concentration of 1 μM in 300 μL media. One hour after adding FITC–dextran, 30 ul medium was taken from the lower chamber. The relative fluorescence unit (RFU) of FITC–dextran was measured by microplate reader at an excitation of 490 nm and emission of 520 nm [47]. Untreated samples served as control. The permeability coefficient (Pc) was calculated according to the equation Pc = (RFU_test_ − RFU_blank_)/(RFU_control_ − RFU_blank_), where RFU_test_ is fluorescence intensity of the treatment samples, RFU_control_ is fluorescence intensity of the untreated samples, and RFU_blank_ is fluorescence intensity of the blank well without any treatment.

### 2.6. Animal and Treatment

Six-week-old C57BL/6J mice were purchased from Orientbio (Seongnam, Korea). One week after acclimation, mice were randomly divided into three experimental groups: control (n = 4), methamphetamine-administration (MA, n = 4), and MA+Y-27632 (n = 4). The mice in the experimental group were given METH (10 mg/kg) by intraperitoneal injection (i.p.) 4 times at 2 h intervals. The control group was administered with saline (i.p.) and Y-27632 (10 mg/kg) was pretreated 1 h before the first METH injection. The mice were sacrificed 3 h after the final METH injection. Experimental procedures and animal care were carried out in accordance with requirements by the Institutional Animal Care and Use Committee at Keimyung University (Daegu, Korea; EXP-IRB number: KM_2023-30; the date of approval: 12 November 2023). The experiments were performed in accordance with the Keimyung University’s scientific research guidelines and regulations.

### 2.7. In Vivo BBB Permeability Assay

BBB permeability was assessed using sodium fluorescein (NaF), as previously described [48,49]. Briefly, mice were injected with NaF (10% in 100 μL PBS), which was allowed to circulate for 1 h. The animals were anesthetized with isofluorane and perfused with PBS. The brains were harvested and homogenized in PBS (1:10 g/v) followed by protein measurement. The samples were then precipitated in 10% trichloroacetic acid overnight at 4 °C. Supernatant was collected after centrifugation at 1000× *g* for 10 min, and then mixed with 5 M NaOH. Fluorescence intensity was detected using a fluorescence plate reader with excitation at 460 nm and emission at 515 nm.

### 2.8. Western Blot Analysis

HBMECs were seeded at a density of 2 × 10^5^ in 6-well plates. After treatment of Y-27632 and METH by the indicated dose and time, cells were washed with cold PBS. Protein of HBMECs was extracted by RIPA buffer (Thermo Fisher, Waltham, MA, USA, NCI9900KR) with protease/phosphatase inhibitor cocktail, and then the concentration of cellular protein was determined by the BCA quantification method. Thirty micrograms from each protein sample was separated by SDS-PAGE and transferred to NC (Nitrocellulose) membrane. The membranes were blocked by 5% BSA in TBS-T for 1 h followed by incubation at 4 °C for overnight of primary antibodies: anti-ZO-1 (1:1000, Invitrogen), anti-VE-cadherin (1:1000, abcam), anti-occludin (1:1000, Invitrogen), anti-claudin-5 (1:1000, Invitrogen), phospho-MLC (1:1000, Cell Signaling Technology, Danvers, MA, USA), phospho-cofilin (1:1000, Cell Signaling Technology), and anti-β-actin (1:1000, Santa Cruz Biotechnology, Dallas, TX, USA). After primary antibody incubation, the membranes were incubated with HRP-conjugated secondary antibody (1:10,000, Santa Cruz Biotechnology) at room temperature for 1 h. Densitometric analyses were performed by using electrochemiluminescence (LAS-3000) [50]. Western blot original images can be found in Supplementary Materials.

### 2.9. Immunofluorescence

HBMECs were seeded onto the collagen-coated coverslips in 24-well plates. After treatment of Y-23762 (10 μM) and fasudil (5 μM) for 30 min followed by METH (1 mM) treatment for 24 h, cells were washed using cold PBS and then fixed with 4% paraformaldehyde for 10 min at room temperature. After washing three times with PBS, cells were blocked by 5% BSA in PBS-T (0.05% Tween) followed by 4 °C overnight incubation of the primary antibodies: anti-ZO-1 (1:200, Invitrogen), anti-VE-cadherin (1:200, abcam), or stained with phalloidin (1:1000), which specifically binds to the F-actin cytoskeleton. Cells were then incubated with secondary antibodies conjugated with Alexa flour 488 or 568 for 1 h at room temperature followed by DAPI staining for nuclear staining. Coverslips were mounted on the glass slides with aqueous mounting solution (Vector Laboratories, Burlingame, CA, USA) and fluorescence images were detected by confocal microscope (LSM5, Carl zeiss, Overkochen, Germany). Three dimensional (3D) plot images were regenerated into the height of fluorescence intensity by using the 3D Surface Interactive Plot plugin tool in ImageJ 1.5i software [51].

### 2.10. RhoA Pull-Down Assay

RhoA pull-down assay was performed with a Rho Activation Assay Biochem Kit (BK036, Cytoskeleton) according to the manufacturer’s instructions. Protein of HBMECs was extracted by cell lysis buffer (50 mM Tris pH 7.5, 10 mM MgCl_2_, 0.5M NaCl, and 2% Igepal) containing protease inhibitor cocktail, and the protein concentration was quantified by BCA assay. Each 500 ug of protein lysates was pulled by 20 ug of rhotekin-RBD beads with a rotator at 4 °C for overnight. After centrifuging for purifying bead-bound-activated Rho A in protein lysates, non-specific bound proteins were removed by washing buffer (25 mM Tris pH 7.5, 30 mM, MgCl_2_, 40 mM NaC). After washing three times with washing buffer, the beads were eluted by 2X lammeli sample buffer and separated in SDS-PAGE. Activated and total RhoA were detected by anti-Rho A (1:1000) and measured by densitometry [52].

### 2.11. Data Analysis

The data were replicated in least three individual experiments and represented as the mean ± standard deviation (SD). Statistical significance was expressed as values of *p* < 0.5, calculated by two-tailed Student’s *t*-test for group comparisons.

## 3. Results

### 3.1. METH Decreased Barrier Integrity Under Non-Toxic Concentration in HBMECs

**Figure 1:**
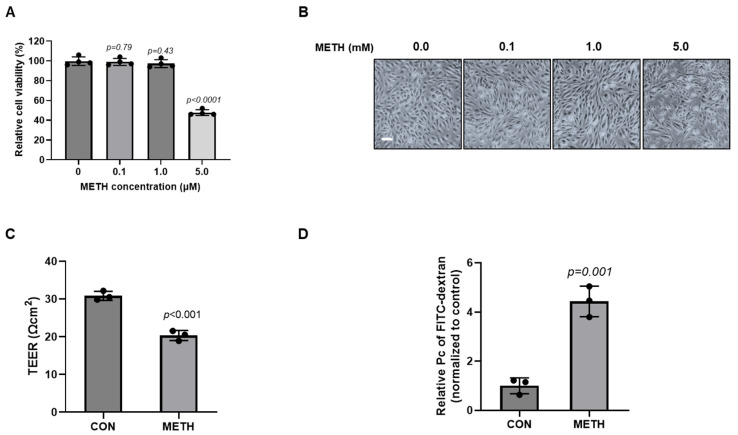
Methamphetamine induces BBB dysfunction in primary human microvascular endothelial cells. (**A**) Cell viability was determined through CCK-8 assay after treatment with 0.1, 1.0, and 5.0 mM METH for 24 h in HBMECs (n = 4). (**B**) Cellular morphology after treatment with METH at the indicated doses was observed under a microscope (scale bar: 100 µm). (**C**) Transendothelial electrical resistance of the HBMEC monolayer was assessed after treatment with METH (1 mM) for 24 h (n = 3). (**D**) Paracellular permeability of the HBMEC monolayer was evaluated by measuring fluorescence intensity of FITC–dextran (70 kDa) after METH (1 mM) treatment for 24 h (n = 3). Data were obtained from three independent experiments in triplicate for each treatment. Untreated samples were designated as control samples. Statistical significance was calculated by two-tailed Student’s *t*-test for group comparisons. All data are presented as mean ± SD.

As described in the Abstract, we used primary human brain microvascular endothelial cells (HBMECs) for in vitro BBB studies in our subsequent experiments. Primarily, in order to determine the non-toxic concentration of METH in HBMECs, we treated the cells with 0.1, 1.0, and 5.0 mM of METH. After treatment with METH at indicated doses for 24 h, we checked the cell viability of HBMECs. While 5 mM of METH exhibited severe toxicity in HBMECs, 0.1 and 1.0 mM METH did not significantly affect cell viability of HBMECs (Figure 1A,B). Consequently, we selected 1 mM concentration of METH for the subsequent studies. Many studies have used paracellular permeability assay and TEER measurement for in vitro BBB function tests [47,50,53]. To investigate the barrier integrity of HBMECs under a non-toxic dose of METH, we performed a permeability assay with FITC–dextran and TEER measurement to evaluate BBB functional integrity. Consistent with previous study, the HBMEC monolayer exhibited baseline TEER values of approximately 30 Ωcm^2^. However, as shown in Figure 1C, METH treatment significantly reduced the electrical resistance in the HBMEC monolayer to approximately 20 Ωcm^2^, indicating a compromised barrier function. In accordance with this result, treatment of METH increased FITC–dextran passage from the luminal chamber to the abluminal chamber, indicating elevated permeability of the HBMECs (Figure 1D). These results showed that METH significantly impairs barrier integrity and enhances the permeability of the HBMEC monolayer under a non-toxic concentration.

### 3.2. METH Disrupts Barrier Integrity and Induces Cytoskeletal Reorganization

**Figure 2:**
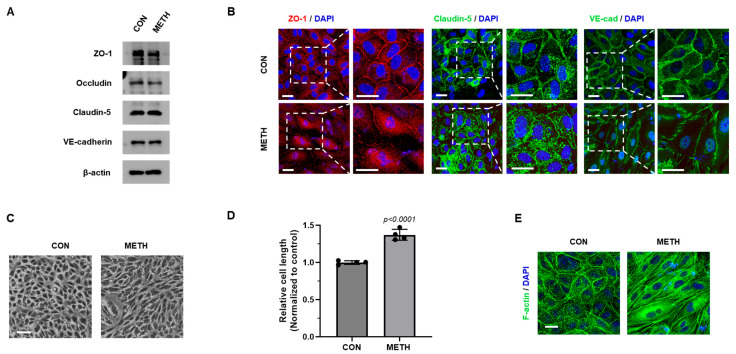
METH induces redistribution of junction proteins and cytoskeletal reorganization in HBMECs. (**A**) Total protein expression of TJ proteins (ZO-1, occluding, and claudin-5) and AJ (VE-cadherin) protein was examined by Western blot after METH (1 mM) treatment for 24 h in HBMECs. (**B**) Confocal microscopy images of ZO-1 (red), Claudin-5 (green), and VE-cadherin (green) were analyzed by immunocytochemistry after treatment with METH (1 mM) for 24 h in HBMECs (scale bar: 20 µm). (**C**) The morphology of HBMECs was captured by a light microscope after METH (1 mM) treatment for 24 h (scale bar: 100 µm). (**D**) Cell length from C (from four random regions with at least 50 cells in each region) was measured using ImageJ 1.5i software. Untreated samples were designated as control samples (n = 4). Statistical significance was calculated by two-tailed Student’s *t*-test for group comparisons. Data are presented as mean ± SD. (**E**) F-actin cytoskeleton was observed by fluorescence microscopy after phalloidin staining following METH (1 mM) treatment for 24 h (scale bar: 20 µm).

It is widely accepted that brain endothelial junctions form the BBB structure, which is a key character to protect brain homeostasis [10]. To investigate whether METH can affect junction proteins of HBMECs, we first examined total protein expression of tight junction and adhesion junction proteins in HBMECs. Compared to the untreated group, the total expression of junction proteins in METH-treated HBMECs did not change at all (Figure 2A). However, we found that METH disrupted junction proteins (ZO-1 and claudin-5) and adherens junction protein (VE-cadherin). Furthermore, we observed that subcellular localization of junction proteins was altered from membrane to cytoplasm (Figure 2B,C). After METH treatment for 24 h, our monitoring showed that cellular morphology of HBMECs became sharper and more stretched (Figure 2D). Cellular morphology is closely related to the actin cytoskeleton [27]. Thus, we checked the F-actin cytoskeleton using phalloidin, which specifically binds to F-actin. While F-actin is located in the peripheral membrane region of non-treated cells, F-actin of METH-exposed cells reorganized, bundled, and elongated across the center of the cells, inducing morphological changes in HBMECs (Figure 2E). These results suggest that METH caused localization changes in junction proteins from the membrane into the cytosol and induces remodeling (bundling/assembly) of F-actin, which reorganizes the cytoskeleton in HBMECs.

### 3.3. Y-27632 Inhibits METH-Induced Activation of Rho A/ROCK Pathway

**Figure 3:**
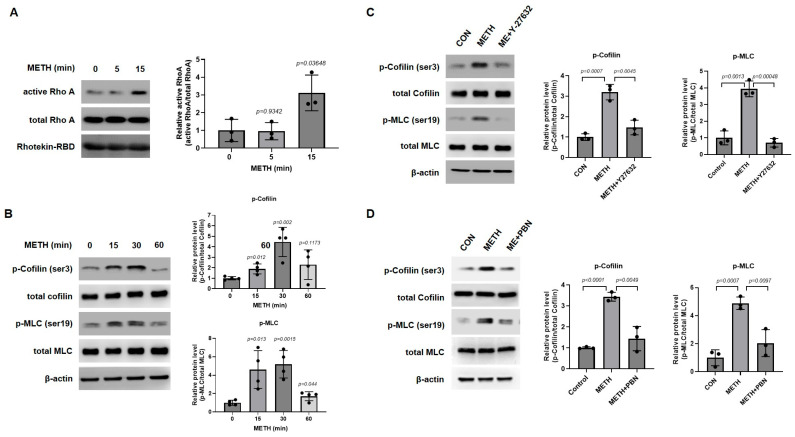
METH-induced RhoA/ROCK signaling pathway activation is inhibited by treatment with Y-27632. (**A**) RhoA activity was measured by RhoA-GTP pull-down assay using Rhotekin-RBD beads. HBMECs were treated with METH (1 mM) for 5 and 15 min (n = 3). (**B**) Phosphorylation level of myosin light chain (MLC) and cofilin in HBMEC treated with METH (1 mM) for 15, 30, and 60 min (n = 4). (**C**,**D**) Detection of p-cofilin and p-MLC was performed by Western blot after pretreatment with Y-27632 (10 µM) or PBN (0.5 µM) for 30 min before METH (1 mM) treatment for 30 min (n = 3). Untreated samples were designated as control samples. Band intensity was analyzed using ImageJ 1.5i software based on least three independent experiments. Statistical significance was calculated by two-tailed Student’s *t*-test for group comparisons. All data are presented as mean ± SD.

It is well established that the activation of the RhoA/ROCK pathway induces a change in cellular morphology through balancing downstream effectors of the RhoA/ROCK signaling pathway [54]. When RhoA is activated by intracellular or extracellular factors, ROCK1 phosphorylates MLC, and ROCK2 activates LIMK, which phosphorylates cofilin [27]. Phosphorylation of cofilin inactivates its function and induces depolymerizing actin filaments, which realigns the cytoskeleton [28]. Since RhoA is activated at a very early time for stimulation [55], we examined whether RhoA activation is stimulated after METH treatment for 5 and 15 min in HBMECs (Figure 3A). Consistent with the previous result, downstream effectors of the RhoA/ROCK pathway, MLC and cofilin, were phosphorylated up to 30 min but decreased at 60 min (Figure 3B). To confirm our data, a commercial ROCK inhibitor, Y-27632, was used to block METH-induced activation of RhoA/ROCK signaling. As shown in Figure 3C, METH induced increased phosphorylation of MLC and cofilin, which were abolished by pretreatment with Y-27632 for 30 min (Figure 3C), suggesting that METH activates the RhoA/ROCK signaling pathway and that Y-27632 inhibits phosphorylation of MLC and cofilin, which are regulators of the cytoskeleton. In our previous study, METH generated excessive reactive oxygen species (ROS), leading to dysfunction in HBMECs [51]. Therefore, we investigated whether ROS is involved in METH-activated RhoA/ROCK signaling. When HBMECs were pretreated with N-tert-butyl-α-phenylnitrone (PBN), a ROS scavenger, the METH-induced increases in MLC and cofilin phosphorylation were attenuated by PBN, suggesting that ROS acts upstream from RhoA/ROCK signaling (Figure 3D).

### 3.4. Inhibition of ROCK Ameliorates METH-Induced Disruption of Junction Proteins Through Cytoskeletal Reorganization

**Figure 4:**
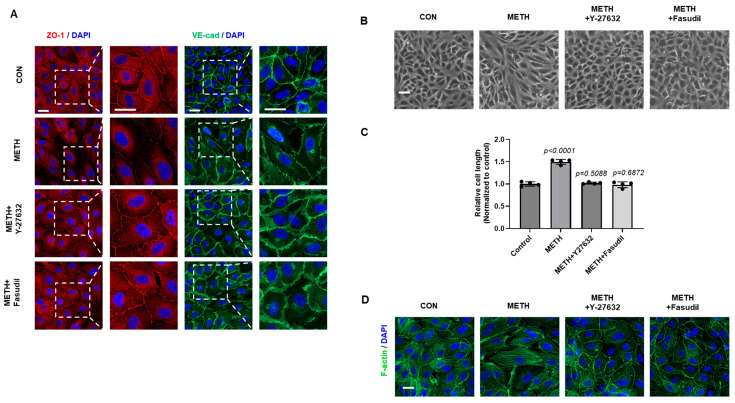
ROCK inhibition alleviates METH-induced redistribution of junction proteins and cytoskeletal rearrangement in HBMECs. (**A**) Fluorescence images of ZO-1 (red) and VE-cadherin (green) were captured by a confocal microscope after treatment of Y-27632 (10 µM) and fasudil (5 µM) followed by METH (1 mM) treatment (scale bar: 20 µm). (**B**) HBMECs were pretreated with Y-27632 (10 µM) and fasudil (5 µM) before METH (1 mM) treatment. Cell morphology was captured under a microscope (scale bar: 100 µm). (**C**) Cell length from B (from four random regions with at least 50 cells in each region) was measured using ImageJ 1.5i software (n = 4). Statistical significance was calculated by two-tailed Student’s *t*-test for group comparisons. All data are presented as mean ± SD. (**D**) F-actin stress fibers were stained by phalloidin after pretreatment with Y-27632 (10 µM) and fasudil (5 µM) followed by METH (1 mM) treatment (scale bar: 20 µm).

Next, we checked whether inhibition of RhoA/ROCK signaling could protect against METH-induced BBB disruption in HBMECs since several studies have reported that ROCK inhibition supressed vascular leakage [39,40,41]. As shown in Figure 4A,B, METH treatment promoted disruption in the membrane distribution of junction proteins, but pretreatment with Y-27632 and fasudil alleviated METH-induced translocalization of TJ proteins and the AJ junction in HBMECs. We also examined cellular morphology and F-actin cytoskeleton in METH-treated HBMECs. After METH treatment, stress fiber formation was observed in line with the result of morphological changes in HBMECs. However, compared with only METH-exposed cells, Y-27632 and fasudil inhibited METH-induced formation of stress fibers, suggesting that these inhibitors prevent METH-induced F-actin cytoskeletal reorganization (Figure 4C,D). These data indicate that ROCK inhibitors attenuate redistribution of junction proteins and cytoskeletal reorganization through the blocking activation of the RhoA/ROCK signaling pathway.

### 3.5. Y-27632 Ameliorates METH-Induced Loss of Functional and Structural Integrity in HBMECs

**Figure 5:**
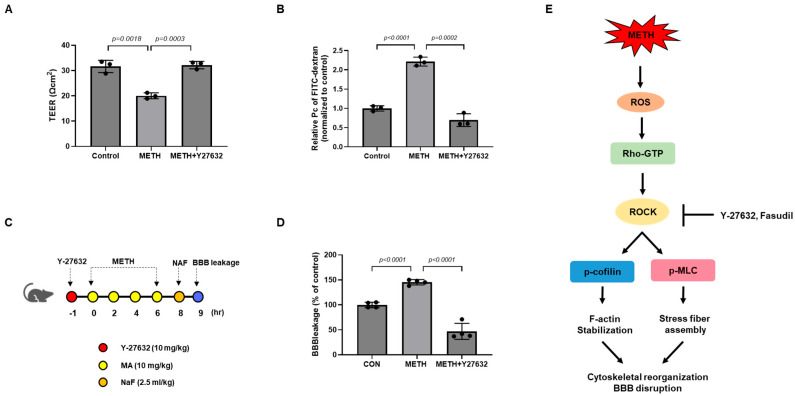
Inhibition of RhoA/ROCK signaling pathway ameliorates METH-induced BBB dysfunction. (**A**) Paracellular permeability was analyzed by FITC–dextran (70 kDa) after treatment with Y-27632 (10 µM) before METH (1 mM) treatment in HBMECs (n = 3). (**B**) Integrity of the HBMEC monolayer was assessed by measuring electrical resistance after pretreatment with Y-27632 (10 µM) followed by METH (1 mM) treatment (n = 3). (**C**) The procedure of the in vivo experiment. METH (10 mg/kg) was administered to mice 4 times at a 2 h interval, and Y-27632 (10 mg/kg) was pretreated 1 h before the first METH injection. (**D**) BBB leakage in METH-administered mice was measured by NaF. Whole brains were used to quantify the leakage of NaF and fluorescence intensity was measured in a microplate reader (Em/Ex 460/515 nm) (n = 4). (**E**) Schematic diagram showing the involvement of the RhoA/ROCK signaling pathway in METH-induced blood–brain barrier disruption. ROCK inhibitors protect HBMECs against methamphetamine-induced blood–brain barrier dysfunction. Statistical significance was calculated by two-tailed Student’s *t*-test for group comparisons. All data are presented as mean ± SD.

To evaluate whether inhibiting RhoA/ROCK signaling could prevent BBB disruption from METH exposure, we first performed a TEER assay, which measures electrical resistance of the endothelial monolayer. METH treatment decreased the monolayer integrity and Y-27632 abolished the METH-induced loss of integrity in HBMECs (Figure 5A). Furthermore, the paracellular permeability of HBMECs was determined by using FITC–dextran. Consistent with the TEER assay data, METH increased the diffusion of FITC–dextran from the luminal chamber to the abluminal chamber, and this effect was alleviated by pretreatment with Y-27632 prior to METH treatment (Figure 5B). Finally, we examined the effect of Y-27632 on BBB protection using a METH-administered mouse model. BBB leakage in the METH-administered mice was measured by sodium fluorescence (NaF). Consistent with in vitro data, Y-27632 ameliorates METH-induced BBB leakage in vivo (Figure 5C,D). These data show the protective effects of ROCK inhibition against METH-induced BBB dysfunction.

## 4. Discussion

Although there are substantial proofs of METH-mediated detrimental effects in neuronal cells, fundamental questions about METH-induced BBB dysfunction still remain largely unanswered. The BBB mainly comprises brain endothelial cells that communicate with neuronal and non-neuronal cells including pericytes, astrocytes, and microglial cells for maintaining CNS homeostasis [56]. Thus, we performed essential experiments using primary HBMECs, which are not only widely utilized but also the most suitable for in vitro work regarding BBB integrity studies and drug discovery related to BBB. In the present study, we highlight that METH exposure elevates paracellular permeability and diminishes barrier integrity of HBMECs through the disruption of junction proteins and cytoskeletal rearrangement, mediated by the activation of the RhoA/ROCK signaling pathway. In addition, we uncovered that the ROCK inhibitor, Y-27632, and fasudil effectively abrogate METH-induced BBB impairment.

Our results demonstrate that METH treatment leads to BBB dysfunction at non-toxic doses, as validated by the cell viability of HBMECs (Figure 1A). Under non-toxic conditions, METH increases HBMEC permeability, which is consistent with decreased integrity of METH-exposed HBMECs (Figure 1C,D). Recent studies have reported that METH destroys endothelial tight connections, which are proved in various brain endothelial cell types: RBE.4 (rat brain microvascular endothelial cells), bEnd.3, and hCMEC/D3 [57,58,59]. Several lines of publications also reported that METH-induced BBB dysfunction causes severe and irreversible brain damage due to endothelial hyperpermeability [19,60]. TJs and AJs are both critical structural proteins that connect adjacent brain endothelial cells to ensure BBB function [11]. In our study, METH treatment destroyed TJ (ZO-1 and claudin-5) and AJ (VE-cadherin) proteins. Unlike a previous study of RBMECs, where METH treatment induced a remarkable decrease in the expression of TJ, including occludin, claudin-5, and ZO-1 [22], our study using HBMECs as an in vitro model showed no difference in protein levels of TJ in response to METH treatment. This dissimilarity may be attributed to species-specific characteristics of the BBB. Although there were no changes in the total expression level of junction proteins, redistributed junction proteins from membrane to cytoplasm in METH-exposed HBMECs were observed (Figure 2). Previous studies suggest that abnormal changes in cytoskeleton could affect intercellular junction proteins through remodeling of actin cytoskeleton, which is anchored to the peripheral tight junction ZO-1 [61]. Thus, our study demonstrates that METH treatment induces the formation of F-actin stress fibers, which are responsible for cellular morphological changes (Figure 2D,E). These results indicate that METH increases paracellular permeability and reduces the barrier integrity of HBMECs through the disruption of junction proteins and the formation of F-actin stress fibers.

To examine the molecular mechanism underlying METH effects on BBB, RhoA/ROCK signaling transduction was investigated. It is well demonstrated that the RhoA/ROCK pathway regulates vascular permeability via TJs and cytoskeleton [25,26]. Activated RhoA activates ROCK, which phosphorylates downstream effector molecules including MLC and cofilin [27]. RhoA/ROCK activation accumulates p-MLC and p-cofilin, which causes abnormal realignment of cytoskeleton induced by excessive assembly of actin filaments [30]. In our study, METH activated RhoA and elevated phosphorylation of MLC and cofilin in HBMECs (Figure 3A,B), consequently leading to the endothelial cytoskeletal reorganization, suggesting the important role of RhoA/ROCK signaling in METH-induced BBB dysfunction.

ROS have been shown to regulate RhoA GTPase through both direct and indirect mechanisms [62,63]. Directly, ROS induces post-translational modifications, such as the reversible oxidation of conserved cysteine residues within the RhoA molecule, particularly at Cys18 in the p-loop. This redox-mediated modification can result in conformational changes that promote nucleotide exchange, leading to RhoA activation [62]. Indirectly, ROS affects upstream regulators of RhoA, such as through the redox-mediated inactivation of low molecular weight protein tyrosine phosphatase (LMW-PTP). This inactivation increases the activity of p190 RhoGAP, which in turn deactivates RhoA [63]. Together, these direct and indirect pathways illustrate the complex role of ROS in modulating RhoA activity in response to oxidative stress. Based on our previous study showing that METH-induced excessive ROS results in dysfunction of HBMECs, we examined whether the RhoA/ROCK pathway is activated by METH-induced ROS. When HBMECs were pretreated with N-tert-butyl-α-phenylnitrone (PBN), a ROS scavenger, PBN attenuated METH-induced phosphorylation of MLC and cofilin in HBMECs, indicating that ROS is upstream from RhoA/ROCK signaling (Figure 5E).

To investigate whether the RhoA/ROCK pathway could serve as a therapeutic target for protecting the BBB against METH-induced damage, we conducted both in vitro and in vivo studies using the ROCK inhibitors Y-27632 and fasudil to evaluate their protective effects. As expected, pretreatment with Y-27632 and fasudil significantly suppressed METH-induced redistribution of TJ (ZO-1) and AJ (VE-cadherin) protein by reducing the phosphorylation of MLC and cofilin (Figure 3C and Figure 4A,B). In addition, METH-induced morphological changes in HBMECs were reversed by pretreatment with Y-27632 and fasudil (Figure 4C). In line with previous data, METH-facilitated F-actin reorganization was also attenuated by Y-27632 and fasudil (Figure 4D). Inhibition of ROCK by Y-27632 treatment reduced paracellular permeability and stabilized BBB structure in METH-exposed HBMECs (Figure 5A,B). Y-27632 also ameliorated METH-induced BBB leakage in vivo, demonstrating that inhibition of ROCK by treatment with Y-27632 and fasudil is effective in protecting the BBB from METH-induced damage (Figure 5C,D).

Recent studies have highlighted the detrimental effects of drug abuse on the functional integrity of the BBB in preclinical models [60,61,62]. Additionally, research has shown that the withdrawal phases from substances such as alcohol, morphine, and METH further aggravate BBB disruption [45,46,63]. Therefore, investigating BBB dysfunction during drug addiction withdrawal and evaluating the potential protective effects of ROCK inhibitors on both BBB integrity and related behavioral traits could provide valuable insights.

## 5. Conclusions

In the present study, we provided that the underlying mechanism of METH-induced BBB disruption is associated with the activation of the RhoA/ROCK signaling pathway. Moreover, Y-27632 and fasudil considerably abolished METH-induced detrimental effects on the BBB through inhibition of ROCK, which phosphorylates downstream molecules, p-MLC and p-cofilin. Further studies are needed to confirm whether the RhoA/ROCK pathway is induced by METH abuse in animal models and, by extension, with METH-developed neurodegenerative diseases. Altogether, our investigation suggests that inhibition of ROCK could be considered as a therapeutic approach for protecting BBB function by targeting the RhoA/ROCK pathway.

## Data Availability

The original contributions presented in the study are included in the article and Supplementary Materials; for further inquiries, please contact the corresponding author.

## Supplementary Materials

The following supporting information can be downloaded at: https://www.mdpi.com/article/10.3390/biom15030340/s1, Original Western Blots.
