# Supplementary material for: Involvement of RhoA/ROCK Signaling Pathway in Methamphetamine-Induced Blood-Brain Barrier Disruption
Source: Biomolecules. 2025 Feb 27;15(3):340. doi: 10.3390/biom15030340 (PMC11940822; doi:10.3390/biom15030340)
Supplement: Supplementary file 1 [file biomolecules-15-00340-s001.zip › biomolecules-3299328-supplementary.pdf]

Rho A signaling pathway regulates methamphetamine-induced BBB dysfunction

Supplementary Figure. Raw data of Western blot bands

Figure 2. ***Hwang et al.***

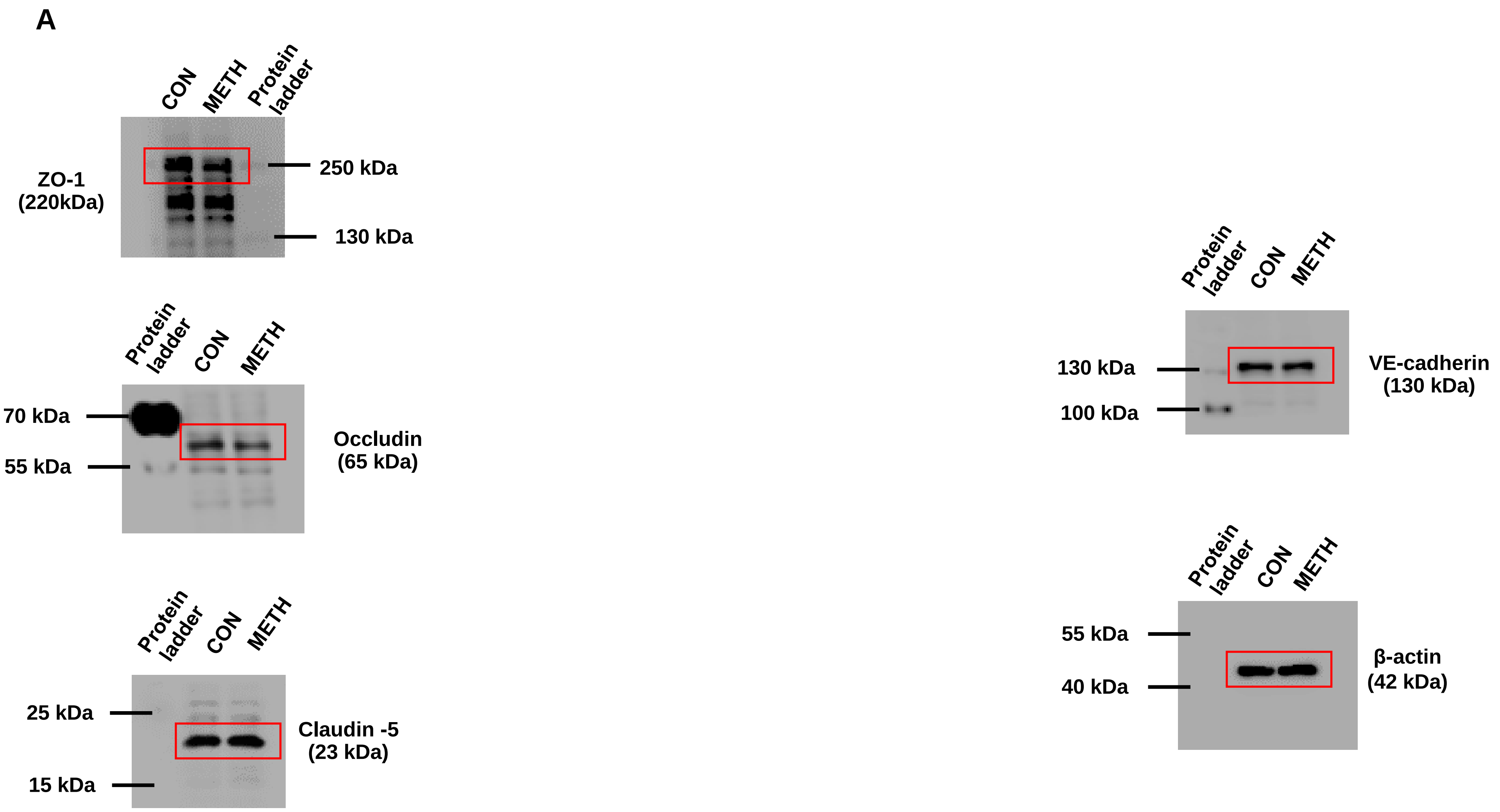

Figure 3. ***Hwang et al.***

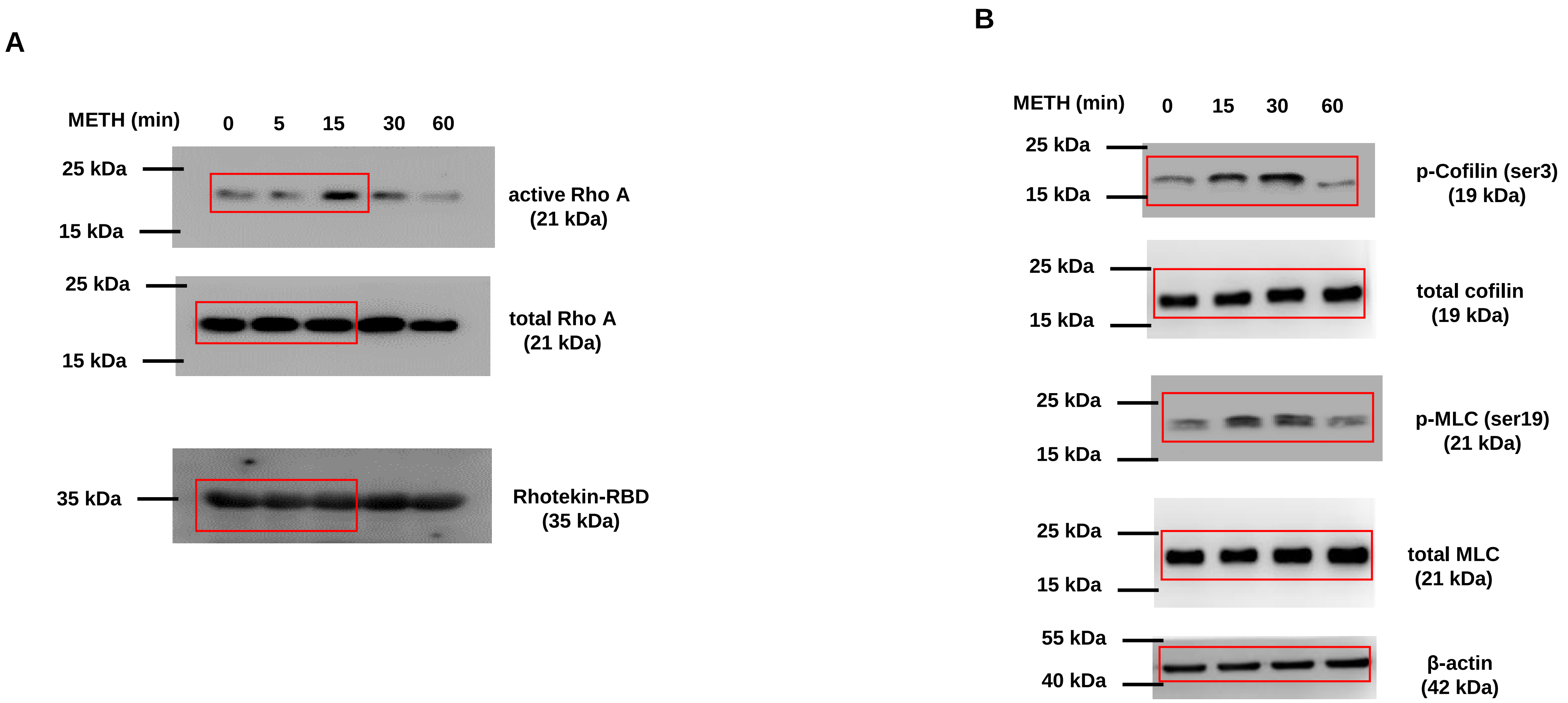

Figure 3. ***Hwang et al.***

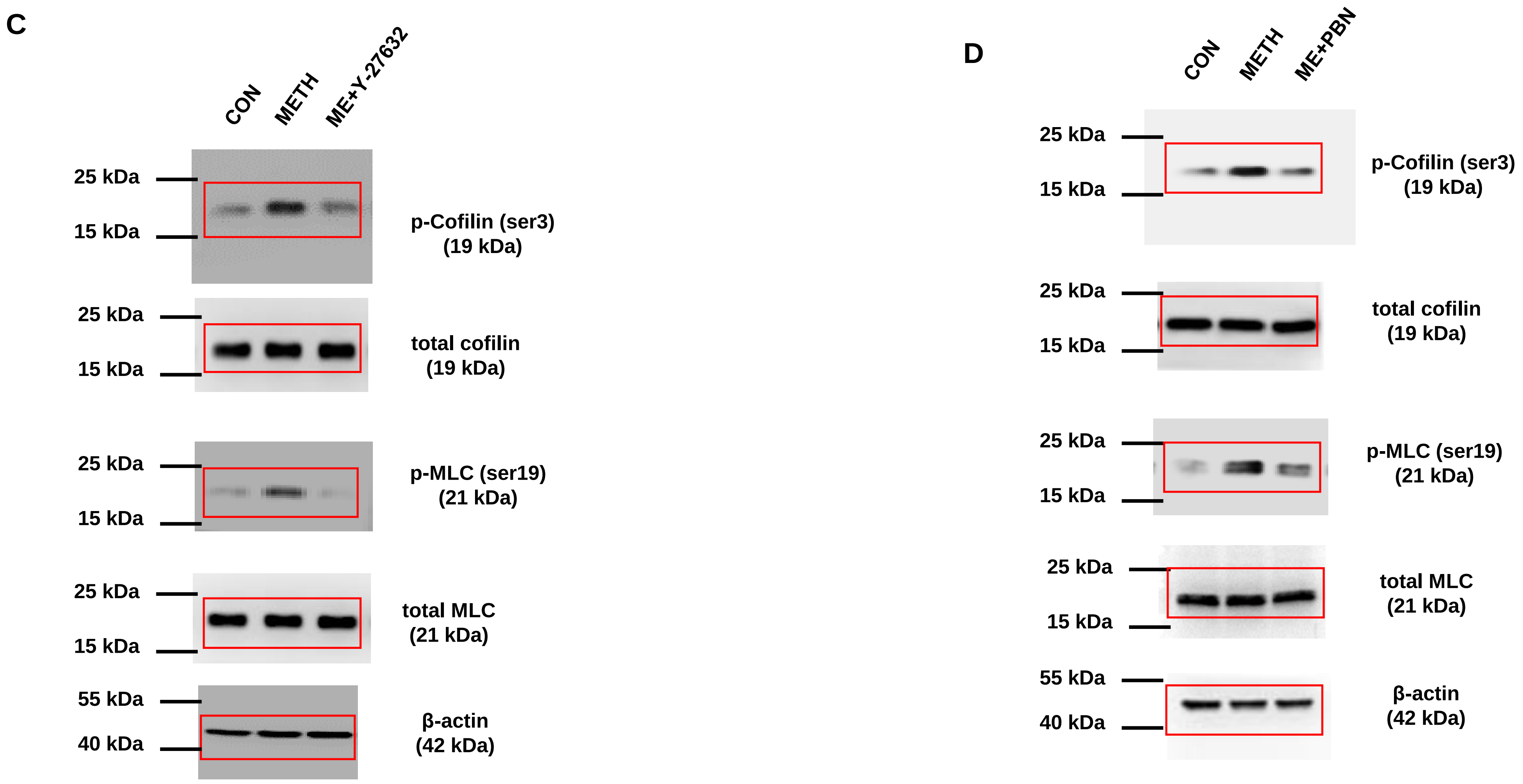

Supplementary Figure. Blots for quantification in Figure 3

Fig. 3A

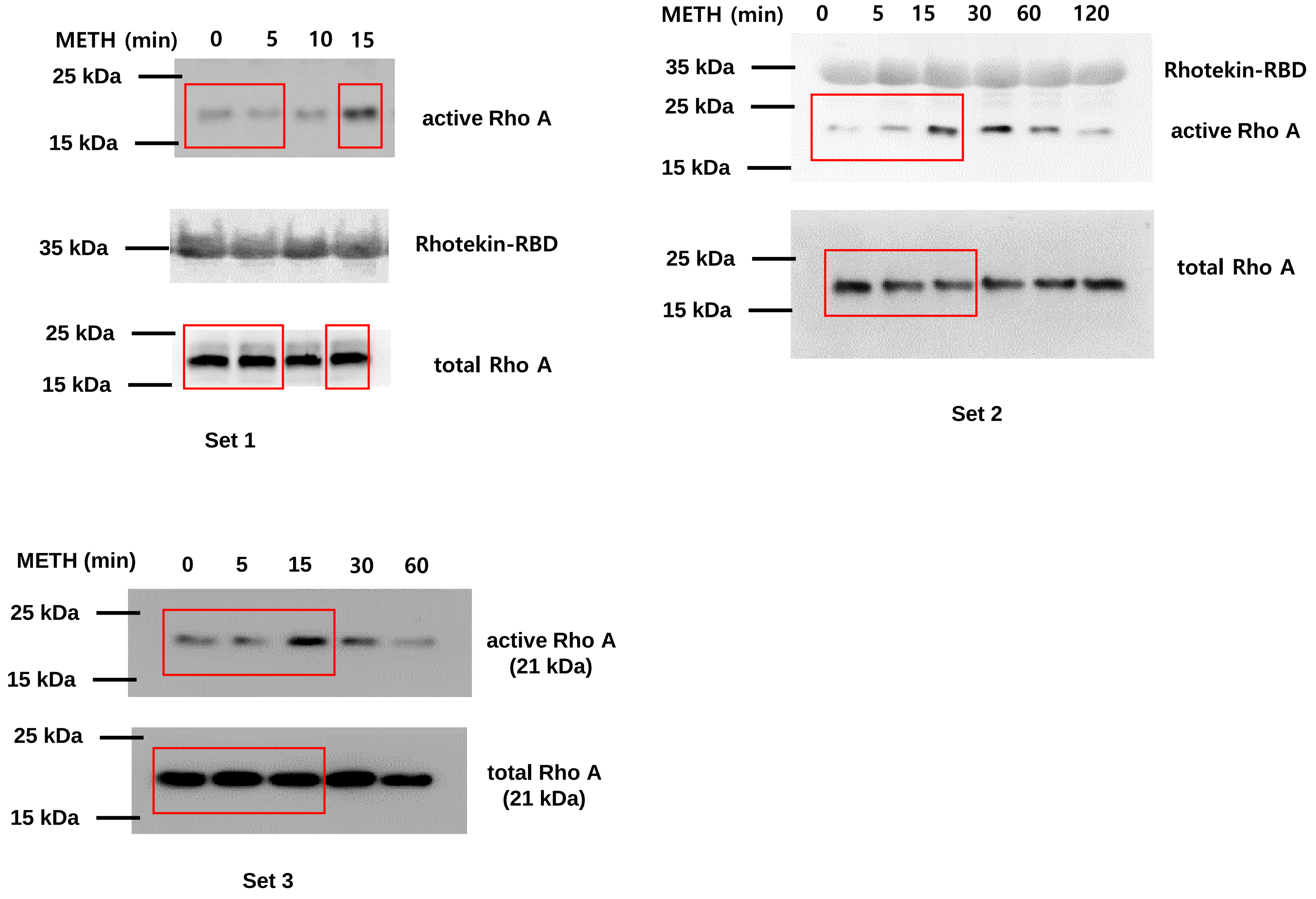
METH (min) 0 5 10 15
25 kDa
15 kDa
active Rho A
35 kDa
Rhotekin-RBD
25 kDa
15 kDa
total Rho A
Set 1
METH (min) 0 5 15 30 60 120
35 kDa
25 kDa
15 kDa
Rhotekin-RBD
active Rho A
25 kDa
15 kDa
total Rho A
Set 2
METH (min) 0 5 15 30 60
25 kDa
15 kDa
active Rho A
(21 kDa)
25 kDa
15 kDa
total Rho A
(21 kDa)
Set 3

Fig. 3B (p-cofilin)

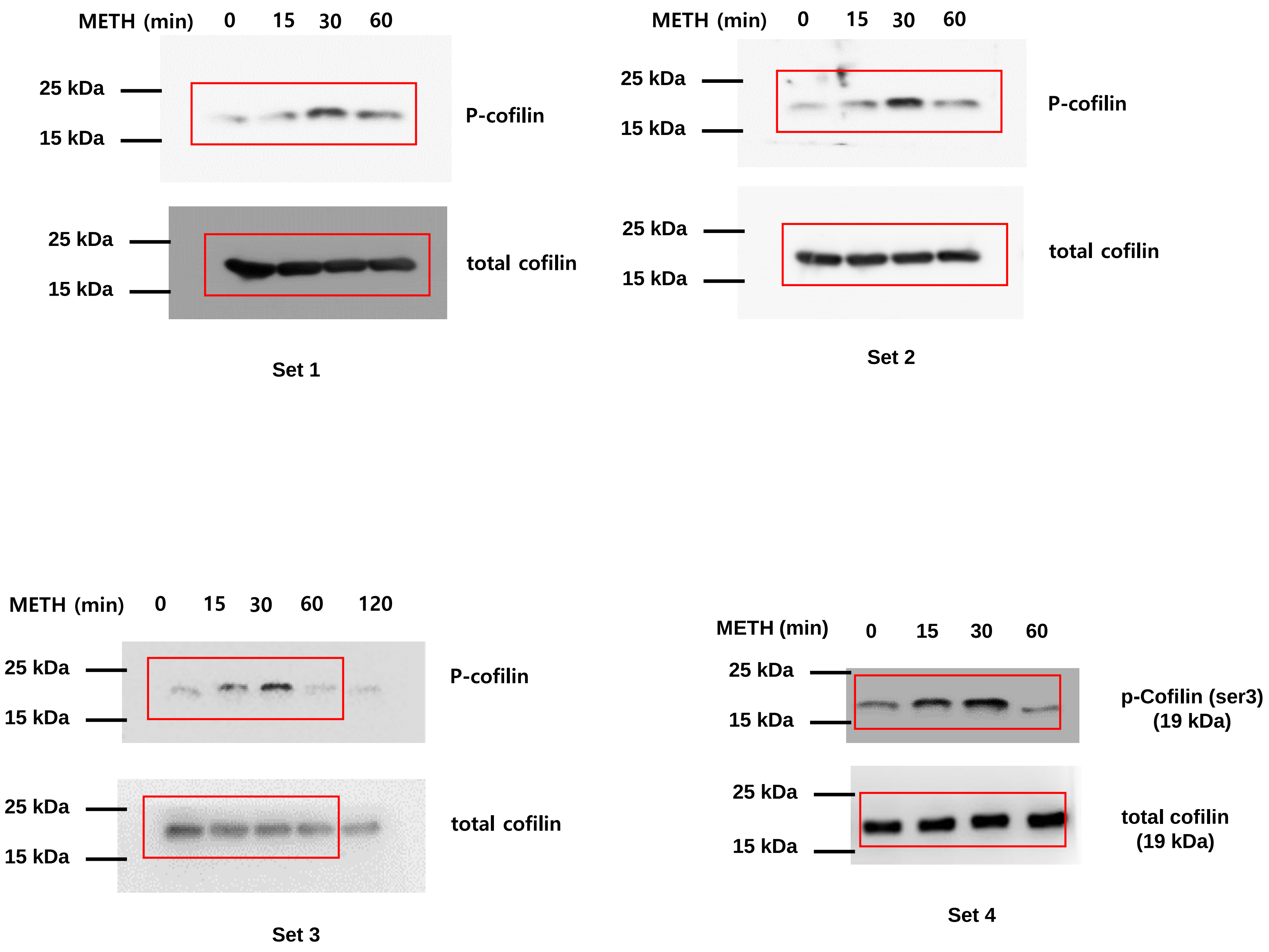

Fig. 3B (p-MLC)

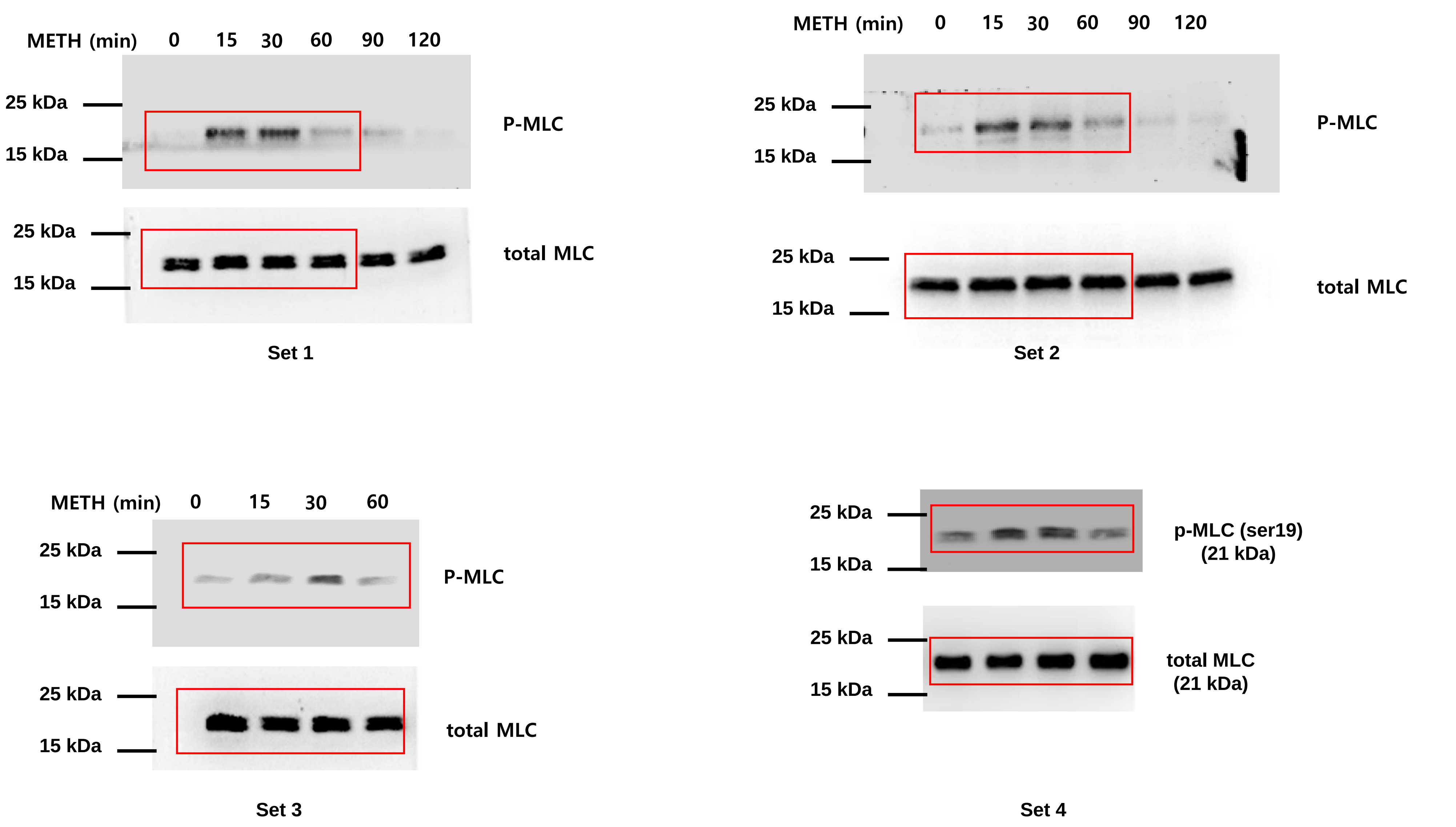
METH (min) 0 15 30 60 90 120
25 kDa
15 kDa
P-MLC
25 kDa
15 kDa
total MLC
Set 1
METH (min) 0 15 30 60 90 120
25 kDa
15 kDa
P-MLC
25 kDa
15 kDa
total MLC
Set 2
METH (min) 0 15 30 60
25 kDa
15 kDa
P-MLC
25 kDa
15 kDa
total MLC
Set 3
25 kDa
15 kDa
p-MLC (ser19)
(21 kDa)
25 kDa
15 kDa
total MLC
(21 kDa)
Set 4

Fig. 3C

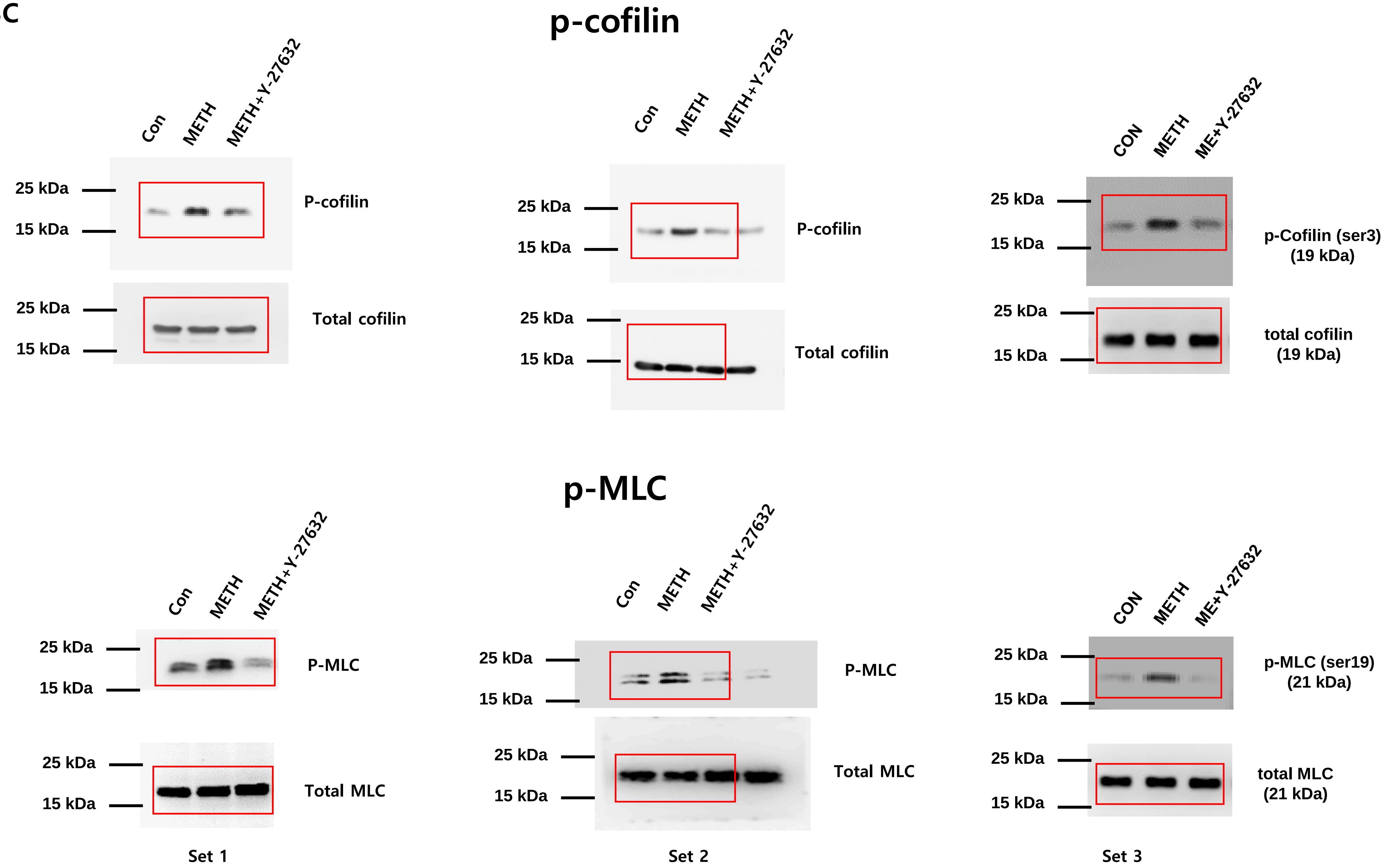
p-cofilin
Con
METH
METH+Y-27632
25 kDa
15 kDa
P-cofilin
Total cofilin
CON
METH
ME+Y-27632
p-Cofilin (ser3)
(19 kDa)
total cofilin
(19 kDa)
p-MLC
P-MLC
Total MLC
p-MLC (ser19)
(21 kDa)
total MLC
(21 kDa)
Set 1
Set 2
Set 3

Fig. 3D

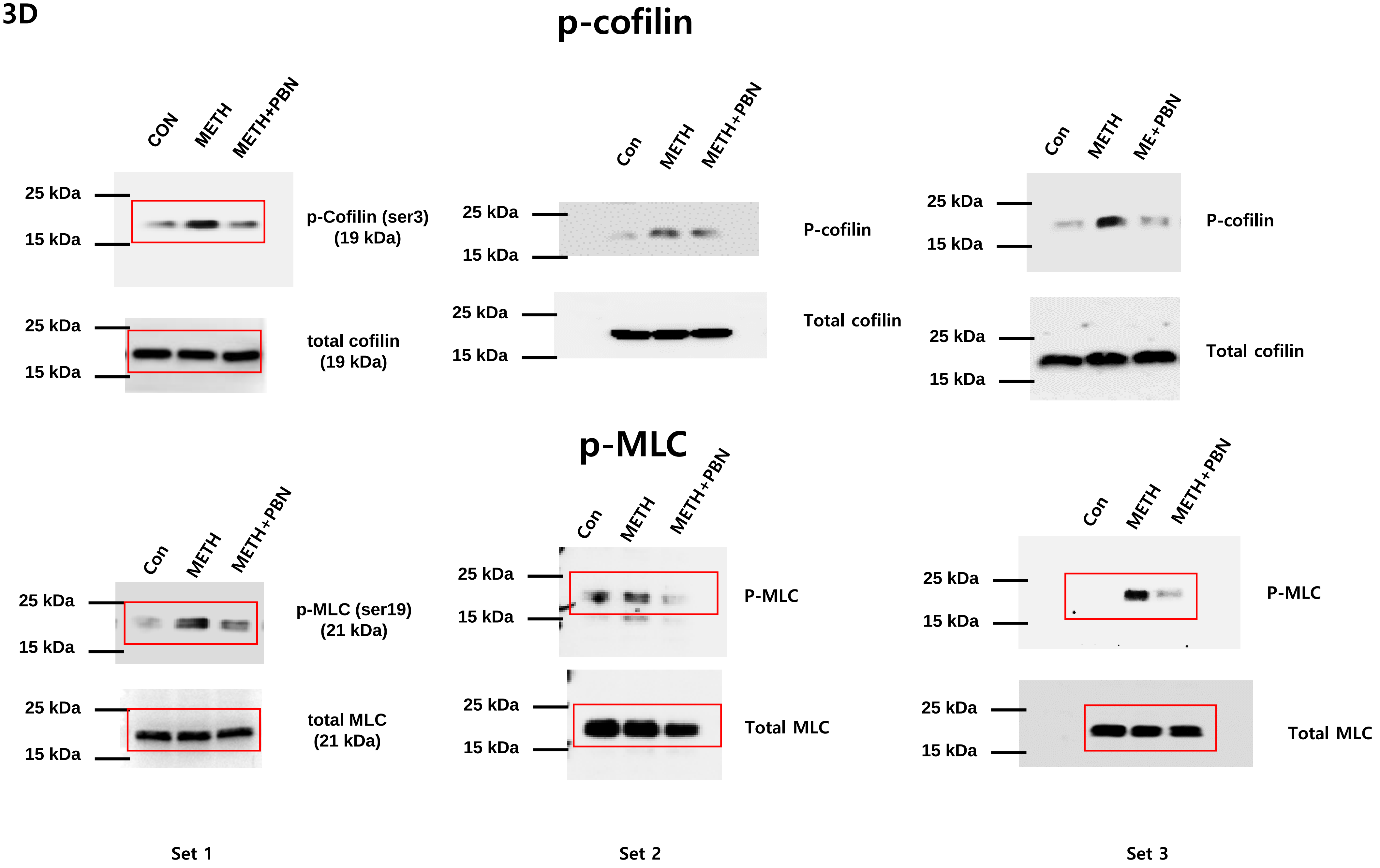
p-cofilin
CON
METH
METH+PBN
25 kDa
15 kDa
p-Cofilin (ser3)
(19 kDa)
25 kDa
15 kDa
total cofilin
(19 kDa)
Con
METH
METH+PBN
25 kDa
15 kDa
P-cofilin
25 kDa
15 kDa
Total cofilin
Con
METH
ME+PBN
25 kDa
15 kDa
P-cofilin
25 kDa
15 kDa
Total cofilin
p-MLC
Con
METH
METH+PBN
25 kDa
15 kDa
p-MLC (ser19)
(21 kDa)
25 kDa
15 kDa
total MLC
(21 kDa)
Con
METH
METH+PBN
25 kDa
15 kDa
P-MLC
25 kDa
15 kDa
Total MLC
Con
METH
METH+PBN
25 kDa
15 kDa
P-MLC
25 kDa
15 kDa
Total MLC
Set 1
Set 2
Set 3
